# Mutation spectrum in *GNAQ* and *GNA11* in Chinese uveal melanoma

**DOI:** 10.1093/pcmedi/pbz021

**Published:** 2019-11-13

**Authors:** Edward D Zhang, Meixia Zhang, Gen Li, Charlotte L Zhang, Zhihuan Li, Guangxi Zang, Zhiguang Su, Ming Zhang, Daoman Xiang, Ling Zhao, Jie Zhu

**Affiliations:** 1 Guangzhou Regenerative Medicine and Health Guangdong Laboratory, Guangzhou 510530, China; 2 Department of Ophthalmology, West China Hospital, Sichuan University, Chengdu 610041, China; 3 Guangzhou Women and Children's Medical Center, Guangzhou Medical University, Guangzhou 510623, China; 4 Molecular Medicine Center, West China Hospital, Sichuan University, Chengdu 610041, China; 5 State Key Laboratory of Ophthalmology, Zhongshan Ophthalmic Center, Sun Yat-sen University, Guangzhou 510060, China

**Keywords:** uveal melanoma, GNAQ, GNA11, somatic mutations, Chinese

## Abstract

Uveal melanoma is the most common intraocular cancer in the adult eye. R183 and Q209 were found to be mutational hotspots in exon 4 and exon 5 of *GNAQ* and *GNA11* in Caucasians. However, only a few studies have reported somatic mutations in *GNAQ* or *GNA11* in uveal melanoma in Chinese. We extracted somatic DNA from paraffin-embedded biopsies of 63 Chinese uveal melanoma samples and sequenced the entire coding regions of exons 4 and 5 in *GNAQ* and *GNA11*. The results showed that 33% of Chinese uveal melanoma samples carried Q209 mutations while none had R183 mutation in *GNAQ* or *GNA11*. In addition, seven novel missense somatic mutations in *GNAQ* (Y192C, F194L, P170S, D236N, L232F, V230A, and M227I) and four novel missense somatic mutations in *GNA11* (R166C, I200T, S225F, and V206M) were found in our study. The high mutation frequency of Q209 and the novel missense mutations detected in this study suggest that *GNAQ* and *GNA11* are common targets for somatic mutations in Chinese uveal melanoma.

## Background

Uveal melanoma is the most common primary malignancy in the adult eye in Western countries.^1^ It is a cancer of the melanocytes in the choroid, ciliary body, and iris, which comprise the uvea. The most frequent (90%) site of uveal melanoma is in the choroid.^2^ Melanoma in the uvea comprises 5% of all melanomas,^3^ and has a particularly strong tendency to metastasize to the liver.^4^

**Table 1:** Frequencies of mutations in R183 (exon 4) and Q209 (exon 5) of *GNAQ* and *GNA11.*

*GNAQ*				*GNA11*				Study
R183 mutations		Q209 mutations		R183 mutations		Q209 mutations		
Number/total	Frequency, %	Number/total	Frequency, %	Number/total	Frequency, %	Number/total	Frequency, %	
0/63	0	7/63	11.1	0	0	14/63	22.2	Present study
4/145	2.8	73/163	44.8	3/145	2.1	52/163	31.9	Ref. [10]

**Figure 1:**
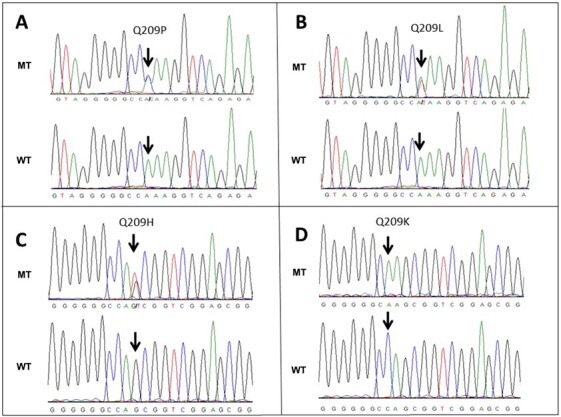
Mutations in Q209 in exons 5 of *GNA11* and *GNAQ*. MT, Mutant; WT, Wild type.

The pathogenesis underlying uveal melanoma is unclear. To date, there are few known risk factors for uveal melanoma, one of which is nevus of Ota, which is an intradermal proliferation of melanocytes that manifests clinically as scleral and periorbital bluish-gray hyperpigmentation.^5^^,^^6^ Uveal melanoma is more commonly found in patients who are older.^4^ Phenotypic features such as light skin and high susceptibility to sunburn, as well as environmental exposure to ultraviolet radiation, are some of the risk factors that have been strongly implicated in the mutagenesis and carcinogenesis underlying cutaneous melanoma; however, the epidemiologic evidence to support the same connection in uveal melanoma remains inconsistent.^7^ Furthermore, a predisposition to uveal melanoma based on race remains unclear, despite the observation that uveal melanoma is much more common in people with light skin compared to those with darker skin.^8^ In Chinese, as in populations from the Western hemisphere, uveal melanoma is the most frequently occurring primary cancer in the adult eye, and was found to be more frequently encountered than previously suggested.^9^

**Figure 2:**
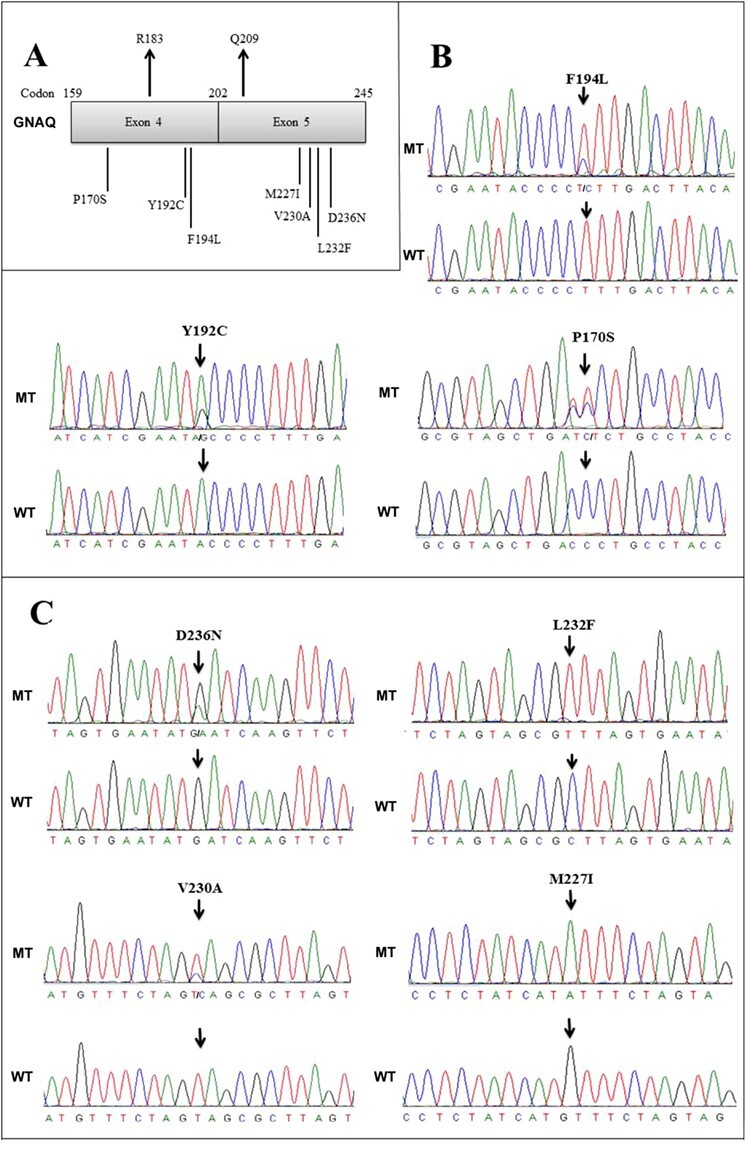
Novel mutations in exon 4 and exon 5 of *GNAQ*. (A) Relative position of mutations in GNAQ. (B) Novel mutations in exon 4. (C) Novel mutations in exon 5. MT, Mutant; WT, Wild type.

**Table 2:** Novel mutations in exons 4 and 5 of *GNAQ* and *GNA11.*

**Sample ID**	**DNA change**	**Codon change**	**Protein change**	**Comment**
	*GNAQ* exon 4			
M6	575A > G	TAC > TGC	Y192C (Tyr192Cys)	Compound heterozygous for a second novel mutation in R166C in *GNA11*
M11	580 T > C	TTT > CTT	F194L (Phe194Leu)	Compound heterozygous for a second mutation in Q209L of *GNA11*
M45	508C > T	CCT > TCT	P170S (Pro170Ser)	Compound heterozygous for a second novel mutation in M227I in *GNAQ*
	*GNAQ* exon 5			
M5	706G > A	GAT>AAT	D236N (Asp236Asn)	Compound heterozygous for a second mutation in Q209P in *GNAQ*
M12	694C > T	CTT > TTT	L232F (Leu232Phe)	Homozygous for this mutation and homozygous for missense mutation Q209P in *GNAQ*
M36	689 T > C	GTA > GCA	V230A (Val230Ala)	
M45	681G > A	ATG > ATA	M227I (Met227Ile)	Homozygous for this mutation, and compound heterozygous with mutation P170S in *GNAQ*
	*GNA11* exon 4			
M6	496C > T	CGC > TGC	R166C (Arg166Cys)	Compound heterozygous for a second novel mutation in Y192C in *GNAQ*
M17	599 T > C	ATC > ACC	I200T (Ile200Thr)	
	*GNA11* exon 5			
M42	674C > T	TCC > TTC	S225F (Ser225Phe)	
M56	616G > A	GTG > ATG	V206M (Val206Met)	

Recent investigation by Van Raamsdonk *et al*. into the genetic predisposition to uveal melanoma has revealed that frequent somatic mutations (R183 or Q209) in *GNAQ* and its paralogous gene *GNA11* are found in 83% of uveal melanomas, and this has established the oncogenic potential of these genes in uveal melanoma.^6^^,^^10^ In light of the finding of these mutational hotspots in *GNAQ* and *GNA11* in Caucasians, we hypothesized that somatic mutations in *GNAQ* or *GNA11* may also be frequently observed in uveal melanoma in the Chinese population. We sequenced and explored the most frequent mutations and novel missense somatic mutations in the entire coding regions of exons 4 and 5 in *GNAQ* and *GNA11* in Chinese uveal melanoma samples.

## Methods

### Participants and clinical examinations

This study used de-identified tumor samples. Patient privacy sensitive information was removed and eye samples anonymized before study. The patients were diagnosed with uveal melanoma by clinical history, standard complete ophthalmic examination, and ancillary study. A total of 63 eyes enucleated from Chinese uveal melanoma patients were paraffin-embedded between 2005 and 2011.

### PCR and DNA sequencing

Somatic DNA samples were extracted from paraffin–embedded uveal melanoma biopsies with a QIAamp DNA FFPE Tissue Kit (Qiagen Inc., Chatsworth, CA, USA) according to the manufacturer’s instructions. The exon 4 and exon 5 of *GNAQ* and *GNA11* were amplified by nest PCR, which was performed in a Bio–Rad T100 thermal Cycler. Using outer set primers (Table S1) and 10ng extracted somatic DNA as template: 1^st^ PCR procedure initial denaturation at 95 °C for 3 minutes, followed by 36 cycles at 95 °C for 30 s, 55 °C for 30 s, and 72 °C for 30 s, then extension at 72 °C for 5 min, and keep at 4 °C for 10 min. Using inner set primers (Table S1) and 1 µL 1^st^ PCR product as template, 2^nd^ PCR procedure is: initial denaturation at 95 °C for 3 minutes, followed by 33 cycles at 95 °C for 30 s, 55 °C for 30 s, and 72 °C for 30 s, then extension at 72 °C for 5 min, and keep at 4 °C for 10 min.

Direct nucleotide sequence analysis was completed for both *GNAQ* and *GNA11*. The exon 4 and exon 5 of *GNAQ* and *GNA11* were amplified by PCR and sequenced on Genetic Analyzer 3130 (Applied Biosystems). Amplified exon 4 and exon 5 of *GNAQ* and *GNA11* were sequenced on Genetic Analyzer 3130 (Applied Biosystems) using one of inner primers (Table S1).

**Figure 3:**
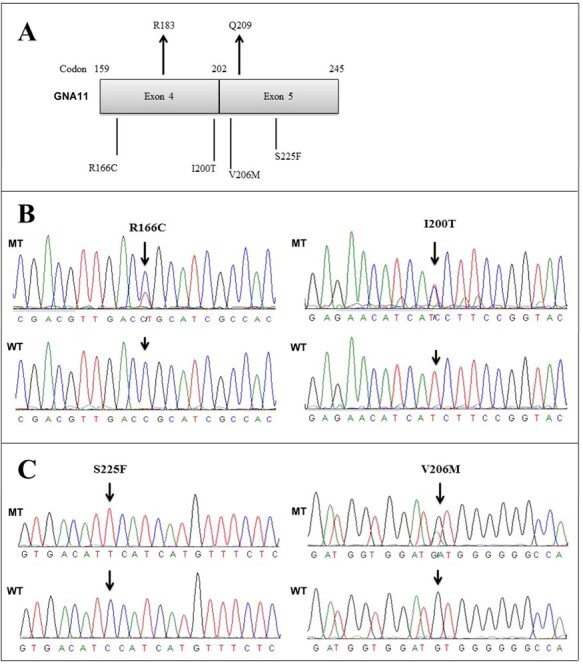
Novel mutations in exon 4 and exon 5 of *GNA11*. (A) Relative position of mutations in GNA11. (B) Novel mutations in exon 4. (C) Novel mutations in exon 5. MT, Mutant; WT, Wild type.

## Results

### Demographic information

Of 63 Chinese samples with primary uveal melanoma, 55.6% were from males. The average age of patients was 51 ± 1.8 years old.

### Mutations in Q209 in exons 5 of *GNA11* and *GNAQ*

Of 63 Chinese samples with uveal melanoma, we found seven somatic Q209 mutations in *GNAQ* (11.1%) and 14 Q209 mutations in *GNA11* (22.2%) (Table 1). This equates to a frequency of 33.3% of samples carrying a Q209 mutation in *GNAQ* or *GNA11*. Among the seven mutations affecting the glutamine codon 209 in *GNAQ*, there were five (71.4%) substitutions for proline (Q209P) and two (28.6%) substitutions for leucine (Q209L) (Fig. 1). Among the 14 mutations affecting the glutamine codon 209 in *GNA11*, there were 11 (78.6%) substitutions for leucine (Q209L), one (7.1%) substitution for histidine (Q209H), one (7.1%) substitution for proline (Q209P), and one (7.1%) substitution for lysine (Q209K) (Fig. 1).

### Mutations in R183 in exons 4 of *GNAQ* and *GNA11*

No somatic mutations in R183 in exons 4 of *GNAQ* and *GNA11* were found in 63 Chinese uveal melanoma samples (Table 1).

### Novel mutations in *GNAQ*

We included the entire coding regions of exons 4 and 5 of *GNAQ* in our sequencing analysis (Fig. 2A). In exon 4 of *GNAQ*, we found three novel somatic mutations: Y192C, F194L and P170S (Table 2, Fig. 2B). Individual M6, in addition to carrying the novel mutation in codon 192, also had a compound heterozygous novel mutation in codon 166 in exon 4 of the paralogous gene *GNA11*. Individual M11 had a novel mutation in codon 194 (F194L) in addition to a compound heterozygous mutation Q209L. Individual M45 had two novel compound heterozygous mutations in *GNAQ*, including P170S in exon 4 and M227I in exon 5.

**Figure 4:**
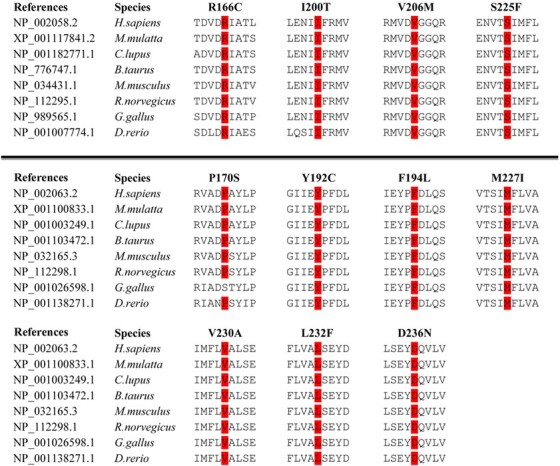
Conservation of the mutations of *GNAQ* and *GNA11*.

In exon 5 of *GNAQ*, we found four novel mutations: D236N, L232F, V230A, and M227I (Table 2, Fig. 2C). Individual M5, in addition to carrying the mutation D236N, also had in the same exon a compound heterozygous mutation Q209P. Sample M12 carried homozygous missense mutations L232F in both alleles of *GNAQ*, and was also homozygous for mutation Q209P in the same exon. Sample M45 carried homozygous mutations V230A in *GNAQ*, in addition to carrying a novel compound heterozygous mutation in P170S in exon 4 of *GNAQ*.

### Novel mutations in *GNA11*

We included the entire coding regions of exons 4 and 5 of *GNA11* in our sequencing analysis (Fig. 3A). In exon 4 of *GNA11*, we detected two novel somatic mutations, including R166C and I200T (Table 2, Fig. 3B). Of note, individual M6 carried a novel mutation in R166C as well as a compound heterozygous novel mutation Y192C in exon 4 of *GNAQ*. In exon 5 of *GNA11*, we detected two novel mutations, including S225F and V206M (Table 2, Fig. 3C).

### Conservation of the mutations

All novel missense mutations detected in exons 4 and 5 of *GNAQ* and *GNA11* were located in revolutionarily conserved regions (Fig. 4).

## Discussion

Uveal melanoma is the most common primary cancer in the adult eye. Based on previous studies, 83% of uveal melanoma carries R183 or Q209 mutations in *GNAQ* or its paralogue *GNA11.* Exon 4 and exon 5 in *GNAQ* and *GNA11* are mutational hotspots in uveal melanomas in Caucasians.^10^

In this study, one-third (33.3%) of 63 Chinese primary uveal melanoma samples carried mutations in Q209 of *GNAQ* or *GNA11*, while the frequency was 76.6% in the Caucasian population (Table 1). Compared to previous studies, we found seven novel missense somatic mutations in *GNAQ* (Y192C, F194L, P170S, D236N, L232F, V230A, and M227I) and four novel missense somatic mutations in *GNA11* (R166C, I200T, S225F, and V206M). The high frequency of Q209 mutations and the large number of novel missense mutations found in this study suggest that somatic mutations in *GNAQ* and *GNA11* are also frequent in uveal melanoma in Chinese people.

Of note, about 4.9% of uveal melanoma samples carried R183 mutations in Van Raamsdonk’s study.^10^ However, R183 mutations were absent in Chinese uveal melanoma samples in our study. The implication of the exclusive absence of this mutation in the Chinese population may need to be validated in a future study with a larger sample size.

*GNAQ* and *GNA11* encode Gα_q_ and Gα_11_, respectively, which make up the alpha subunits of heterotrimeric G proteins. The alpha subunit performs the essential GTPase function of hydrolyzing a GTP molecule and thereby turning off the signaling mechanism between membrane-bound G-protein-coupled receptors and their intracellular downstream effectors.^11^^,^^12^ Mutations that substitute amino acid residues at the GTP contact site, including glutamine at codon 209 and arginine at codon 183, were found to transform *GNAQ* and *GNA11* into oncogenes and induce the constitutive activation of the mitogen-activated protein (MAP) kinase pathway.^13–15^ Recently, it was predicted that constitutive activation of Gq or G11 in uveal melanomas results in abnormal Yes-associated protein (YAP) activation, which contributes to uveal melanoma development.^16^^,^^17^

There is abundant evidence that uveal melanoma and cutaneous melanoma are distinct tumors.^18^^,^^19^ Uveal melanoma and cutaneous melanoma differ significantly in their epidemiological, clinical, immunophenotypical, and cytogenetic characteristics.^20^*BRAF* and *NRAS* are common mutation targets in cutaneous melanoma.^21^^,^^22^ However, it has been reported that mutations in *BRAF* and *NRAS* are absent in uveal melanoma.^23^^,^^24^

Clinically, uveal melanoma constitutes a grave diagnosis because of the risk of vision loss and high potential for metastatic disease, which is almost always fatal. Current therapy includes plaque brachytherapy, proton beam radiotherapy, transscleral local resection, transpupillary thermotherapy, and enucleation. Recently, the use of inhibitors of MAPK pathway and its downstream effectors has shown promise in treating *GNAQ* mutant uveal melanoma.^6^^,^^25^^,^^26^

In conclusion, this study found that somatic mutations in *GNAQ* and *GNA11* occur frequently in uveal melanoma in Chinese people. The recognition of novel genetic aberrants, especially those in mutational hotspots of uveal melanoma, is an ongoing effort toward a more complete genetic understanding of this disease and may aid discovery of effective clinical therapies.

## Supplementary Material

Table_S1_pbz021
