# Supplementary material for: Mutation spectrum in GNAQ and GNA11 in Chinese uveal melanoma
Source: Precis Clin Med. 2019 Nov 13;2(4):213–20. doi: 10.1093/pcmedi/pbz021 (PMC8985776; doi:10.1093/pcmedi/pbz021)
Supplement: Table_S1_pbz021 [file table_s1_pbz021.docx]

**Table S1.** **Primers used for PCR and sequencing**

| Gene | Sequence (5’-3’) |
| --- | --- |
| GNAQ-Exon4-outer-F | GACTCCTCTACCACTTTCTGAT |
| GNAQ-Exon4-outer-R | GAAGCCTACACATGATTCCAGT |
| GNAQ-Exon4-inner-F | GTCCTTCCCTTTCCGTAGACAGCT |
| GNAQ-Exon4-inner-R | GAAGCCTACACATGATTCCAGT |
| GNAQ-Exon5-outer-F | GATCATCGTCATTCAAGAGAAT |
| GNAQ-Exon5-outer-R | GACAGAAGAGCTTACCACAGGATT |
| GNAQ-Exon5-inner-F | CCTAAGTTTGTAAGTAGTGCTAT |
| GNAQ-Exon5-inner-R | GACAGAAGAGCTTACCACAGGATT |
| GNA11-Exon4-outer-F | GGTCCACCCCCTCCTGGTGGCT |
| GNA11-Exon4-outer-R | GATATGAGGTCTGGCTATGTT |
| GNA11-Exon4-inner-F | GGTCCACCCCCTCCTGGTGGCT |
| GNA11-Exon4-inner-R | GTTGCCCAGGGTGGTCTCAAACT |
| GNA11-Exon5-outer-F | GCCGTCCTGGGATTGCAGATT |
| GNA11-Exon5-outer-R | GAGTTCTGGAACCAGGGGTAGGT |
| GNA11-Exon5-inner-F | GCCGTCCTGGGATTGCAGATT |
| GNA11-Exon5-inner-R | GCTTGGCAGGTGGGGAAGGC |
